# Mini-Review: Can the Metastatic Cascade Be Inhibited by Targeting CD147/EMMPRIN to Prevent Tumor Recurrence?

**DOI:** 10.3389/fimmu.2022.855978

**Published:** 2022-03-28

**Authors:** Michal A. Rahat

**Affiliations:** ^1^ Immunotherapy Laboratory, Carmel Medical Center, Haifa, Israel; ^2^ Ruth and Bruce Rappaport Faculty of Medicine, Technion-Israel Institute of Technology, Haifa, Israel

**Keywords:** metastatic cascade, epithelial-to-mesenchymal transition (EMT), disseminated tumor cell (DTC), dormancy, tumor-associated macrophages (TAMs), CD147/EMMPRIN

## Abstract

Solid tumors metastasize very early in their development, and once the metastatic cell is lodged in a remote organ, it can proliferate to generate a metastatic lesion or remain dormant for long periods. Dormant cells represent a real risk for future tumor recurrence, but because they are typically undetectable and insensitive to current modalities of treatment, it is difficult to treat them in time. We describe the metastatic cascade, which is the process that allows tumor cells to detach from the primary tumor, migrate in the tissue, intravasate and extravasate the lymphatics or a blood vessel, adhere to a remote tissue and eventually outgrow. We focus on the critical enabling role of the interactions between tumor cells and immune cells, especially macrophages, in driving the metastatic cascade, and on those stages that can potentially be targeted. In order to prevent the metastatic cascade and tumor recurrence, we would need to target a molecule that is involved in all of the steps of the process, and evidence is brought to suggest that CD147/EMMPRIN is such a protein and that targeting it blocks metastasis and prevents tumor recurrence.

## Introduction

Recurrence of cancer due to a metastatic spread to remote organs is responsible for the vast majority of cancer-related deaths, despite successful treatment of the primary tumor (1). The metastatic cells are plastic, shift between different states and are often more resistant to different modalities of existing treatments (2), and therefore, present a great therapeutic challenge. Solid tumors can metastasize to a regional lymph node or to a secondary organ *via* lymphatics or blood vessels very early in their development, even before diagnosis of the primary tumor (3, 4). Metastasis is a multi-step process that begins with a change in the epithelial phenotype of the cells, a process known as the Epithelial-To-Mesenchymal Transition (EMT). EMT promotes the detachment of some tumor cells, increases their motility and changes their phenotype into a mesenchymal, spindle-like morphology (5). This increases their invasiveness and ability to resist different drugs (6, 7). The increased motility allows a cell to pave its way through the extracellular matrix (ECM), intravasate into a lymphatic or blood vessel to become a circulating tumor cell (CTC), and then extravasate in a regional lymph node or a distant tissue as a disseminated tumor cell (DTC) (8). However, while every metastasizing cell must undergo EMT, it is important to remember that EMT is not a binary process, and cancer cells are found in many hybrid forms along the epithelial/mesenchymal axis (hybrid E/M cells), having both epithelial and mesenchymal features that endow them with the plasticity to adjust to different microenvironments (5, 9). The DTCs that are lodged in the remote organ, either as single cells or as clusters of a few cells, may remain in a latent or quiescent state for years or decades (dormancy), undetected by imaging techniques, until they suddenly start proliferating (colonization). At this stage they undergo the inverse process to EMT - Mesenchymal to Epithelial Transition (MET), that allows their rapid outgrowth (the metastatic outbreak), ultimately generating the metastatic lesion (10).

This multi-step process, known as the metastatic cascade (11), is highly inefficient, and only few of the CTCs that embark on this journey will eventually be implanted in a secondary organ (about 0.01%) and become DTCs (12, 13). Dormant cells have been reported in many types of cancer (e.g., melanoma, lung cancer, glioblastoma, breast cancer, multiple myeloma, leukemia, colorectal, prostate, skin and gastric cancer), and the combination of their early dissemination and resistance to common modalities of treatment increases the risk of tumor recurrence (14, 15). Hence, a major therapeutic goal is to find successful therapies directed against different steps of the metastatic cascade, thus preventing recurrence and metastatic outbreak.

The EMT process initiates the metastatic cascade, and renders tumor cells more resistant to different therapies (16, 17), such as chemotherapies (18), targeted therapies (19) and immunotherapies (20). The EMT process has been linked to resistance to therapy, and several mechanisms were described, some of which are presented here. EMT depends on the induction of the EMT transcription factors (EMT-TFs), particularly members of the Snail, Twist1, and Zeb families. In addition to regulating the epithelial or mesenchymal phenotypes, these EMT-TFs also regulate the expression of ABC transporter genes and genes involved in drug-induced apoptosis, thus contributing to acquired drug resistance (17, 19). For example, high Snail levels induce the activity of the Multidrug Resistance Protein 1 (MDR1)/P-Glycoprotein, thus allowing better transport of drugs outside the cells and improving cell resistance (21), Twist1 suppresses the expression of the pro-apoptotic protein BIM (*BCL2L11*) to mediate resistance to tyrosine kinase inhibitor erlotinib (22), and Zeb1 enhanced the expression of Ataxia-Telangiectasia Mutated (ATM) kinase that promotes the DNA damage repair machinery and confer resistance to agents such as epirubicin (23). The pan-resistance in dormant cells, which inherently exhibit reduced metabolism and arrested proliferation that endows them with some resistance to therapy, is also partially attributed to low-level expression of E-cadherin that upon ligation provides signals that activate ERK and PI3K signaling pathways to ensure survival of DTCs. When these cells are exposed to chemotherapies these signals are amplified leading to drug resistance (2, 15, 24). To promote chemoresistance, extracellular vesicles (Evs) were shown to directly sequester drugs, either by up-taking them and secreting them outside the cells, or by exhibiting receptors or decoy receptors that bind therapeutic antibodies (e.g., with rituximab and CD20 or Herceptin and Her2). EVs can also horizontally transfer proteins, mRNAs, miRNAs or LncRNAs that express or regulate the expression of transporters and transcription factors, from chemo-resistant cells to chemo-sensitive cells or from stroma and immune cells to tumor cells promoting acquired resistance [reviewed in (25)]. The EMT process also promotes the recruitment of macrophages, MDSCs and fibroblasts to the tumor microenvironment (TME), and the tumor cells then interact with them and reprogram them *in situ* (26) to become tumor-associated macrophages (TAMs) and cancer-associated fibroblasts (CAFs) that secrete pro-inflammatory and pro-angiogenic mediators that affect drug resistance as well (16, 19). For example, IL-6 and Oncostatin-M have been implicated in the resistance to cisplatin and gemcitabine, respectively (27, 28). The suppressive microenvironment and increased IFNγ secretion that leads to increased PD-L1 expression on tumor cells and macrophages, inhibit T cells and NK cells and promote immune evasion of the DTCs (29, 30). Lastly, elevated deposition of ECM proteins and expression of integrins, increasing density and stiffness, was associated with increased drug resistance (31, 32).

Targeting the EMT process may enable us to reverse these effects and reduce metastasis. Different approaches to targeting EMT include inhibiting signals that promote it (e.g., TGFβ, HGF), targeting stromal cells that are needed to activate the EMT program, targeting specific EMT transcription factors or targeting the microRNAs that regulate them. Such approaches are extensively reviewed elsewhere (33), but although they may inhibit the metastatic cascade and reduce metastasis, it is not clear how they might affect dormant DTCs that have already disseminated to remote organs. However, dormant DTCs have already undergone EMT and activated dormancy programs (see below) that endow them with increased survival and pan-resistance to different therapies (34), suggesting that eradicating these dormant cells will prove challenging. Another option of targeting CTCs on route to the metastatic site was proposed. CTCs may be detected using liquid biopsies, but their presence after successful treatment or primary tumor resection, might indicate that an already existing dormant tumor sheds its cells (35), and therefore, they are not an obvious target.

There are three strategies to inhibit the outgrowth of dormant DTCs: a) kill DTCs directly, b) awaken dormant cells and simultaneously target them with therapies designed against proliferating cells (e.g., chemotherapies), or c) impose and maintain their dormant state indefinitely, or at least for a long time (36). Killing dormant DTCs or awakening them are strategies that should take into account their inherent resistance to cytotoxic drugs, targeted therapies and even immunotherapies (37, 38). Therefore, if any cells survive treatment, they are likely to become even more aggressive, and promoting their exit from dormancy might expedite their uncontrollable proliferation and enhance tumor relapse. The option of maintaining dormancy for long periods allows tumor cells to remain viable, and risks the possibility that eventually they will escape dormancy, suggesting that treatment should be given for life, thus increasing its potential toxicity and costs.

## What Is Dormancy?

A closer look at dormancy shows that while most colonizing DTCs undergo apoptosis and die, and a few immediately proliferate to generate macro-metastases in a favorable pre-metastatic niche, some DTCs activate survival programs and enter the dormant state. These cells exhibit growth arrest and will remain suspended in the G_0_ phase (cellular dormancy), allowing them to re-enter cell cycle once conditions are more favorable (3, 12). Other DTCs form small cellular clusters that balance proliferation and apoptosis, remaining dormant either because pro-angiogenic signals are lacking (angiogenic dormancy) or because of the pressure exerted by the immune system (immune-mediated dormancy) drives apoptosis of the cells that is balanced by their proliferation (3, 12). The triggers that DTCs encounter in the metastatic microenvironment (MME) that determine their entry into or escape from a dormant state can be divided into cell-intrinsic and cell-extrinsic mechanisms, and both are not yet fully elucidated.

Cell-intrinsic mechanisms include metabolic pathways, autophagy, epigenetic mechanisms, and genetic mutations that directly or indirectly regulate the cell cycle machinery and proliferation. Experiments *in vitro* demonstrated that tumor cells can enter dormancy to survive deprivation of glucose or growth factors (e.g., IGF-2, PDGF) (37). This triggers arrest of proliferation and reduced expression of proteins involved in the regulation of cell cycle (e.g., c-Myc, Cyclin D1), or increased expression of the Cyclin Dependent Kinases (CDKs) inhibitors (e.g., p16, p21, p27) (39). Metabolic pathways respond to stress signaling, and reduction in PI3K signaling or increased autophagy has been linked to dormancy (40). An increase in the ratio between phosphorylated p38 and ERK MAPKs, reflecting stress-induced p38 activation, has often been used as a marker of dormancy, as was the increased expression of the orphan nuclear receptor (NR2F1) which induces SOX9 and RARβ, that in turn, elevate the CDK inhibitors p16 and p27 (3, 39, 41). Therapies that target specific elements in those pathways could be employed to induce dormancy. For example, genetic ablation of the FBXW7 protein that is part of an E3 ligase complex, promotes degradation of Cyclin E and c-Myc (42), whereas inhibitors of autophagy (e.g., hydroxychloroquine) inhibit proliferation and reduce DTCs survival (43). Such approaches are reviewed in (3, 39, 40).

Extrinsic factors in the MME can enforce DTCs dormancy. High levels of quiescent signals can be secreted from organ cells and resident immune cells, such as TGFβ2 and BMP7 in the bone, or BMP4 in the lung (12, 44–46). Thrombospondin-1 (Tsp-1) secreted from quiescent endothelial cells promotes dormancy, whereas in sprouting endothelial cells, TGFβ1 and Periostin secreted from endothelial tip cells promote tumor cell outgrowth (46). Low oxygen tensions (hypoxia) can impair the mTOR metabolic pathway to induce dormancy (47), or induce the expression of TGFβ2 and dormancy marker genes (48, 49). The composition of ECM proteins and the degree of tissue stiffness can also induce dormancy or outgrowth (44, 50, 51). For example, activation of β1-integrins promoted Src-FAK-ERK-MLCK signaling and escape from dormancy (52), and crosslinking of Collagen fibers by Zeb1-regulated Lysyl Oxidases that increase stiffness, promotes metastasis (53). In mouse syngeneic or xenograft models that were treated for oncogene inhibition (anti-Her2 and anti-ER treatments), primary tumors regressed leaving residual tumor cells locally or at remote sites, that exhibited reduced proliferation and a gene signature with reduced expression of Cyclins and CDKs, enhanced expression of ECM proteins (e.g., Fibronectin) and dormancy genes, such as *Bhlhe41* (coding for DEC2), TGFβ2 and its receptor TGFβRIII, and *Thsb1* (coding for Tsp-1). This gene signature was similar to dormancy induced by microenvironmental cues, and was different from genes expressed by primary tumor cells or recurrent metastatic tumor cells (38).

The MME consists of many factors arriving from systemic factors, extracellular vesicles (EVs) secreted from the primary tumor, soluble factors secreted from the resident tissue or immune cells, and from the DTCs themselves. These prepare the pre-metastatic niche (‘soil’) to receive the metastatic cells (‘seeds’) (54), determine organotropic metastasis (affinity of DTCs to specific organs) (55–57), and promote tumor cell-stroma interactions (56, 57) that eventually promote the escape of the DTCs from dormancy. However, the mechanisms that allow these myriad factors and pathways to interact and to determine dormancy or escape from it are only now beginning to be addressed. The reader is referred to some outstanding reviews that discuss these mechanisms and potential approaches to target them (3, 10, 34, 36, 39, 40, 45, 58, 59).

## Macrophages in Metastasis

The central role tumor-associated macrophages (TAMs) play in promoting the primary tumor is now well recognized, but their role in metastasis is only now beginning to unfold. TAMs can be differently activated, and in the primary tumor they are often found in a mixture of both M2-activated (pro-tumoral, pro-angiogenic) and M1-activated (pro-inflammatory, anti-tumoral) macrophages. In the primary tumor, TAMs and myeloid-derived suppressor cells (MDSCs) secrete growth factors and pro-angiogenic factors that promote tumor progression, and express inhibitory receptors and cytokines that suppress cytotoxic T cells or lead to their exhaustion, thus contributing to evasion from immune recognition (60–64).

Crosstalk between specific TAMs subsets and tumor cells in specific niches in the primary tumor also help promote metastasis. Such interactions promote the EMT process and increase the ability of tumor cells to migrate and invade (65–67). Perivascular TAMs (identified as Tie2^+^VEGFA^+^MRC1^+^) promote angiogenesis and vascular permeability by enhancing VEGF secretion (68, 69), and assist in generating specialized temporal structures (called tumor microenvironment of metastasis-TMEM) that are based on direct cell-cell contacts with both tumor cells and endothelial cells. These TMEMs become the gateways which facilitate the intravasation of the metastatic cells (11, 70–72). Another subset of TAMs co-migrate along with the metastatic cells, secrete matrix metalloproteinases (MMPs) that degrade the ECM, and thus pave the way for the metastatic cells (54). TAMs can also utilize a myriad of mechanisms (e.g., secretion of IDO1, TGFβ1, PGE_2_ and IL-10 that promote Tregs, or expression of co-inhibitory receptors as PD-L1 that lead to T cell exhaustion) that provide local protection for the tumor cells against immune recognition along the metastatic cascade (71).

Metastasis-associated macrophages (MAMs) in the distant organs consist of either resident macrophages or blood monocytes-derived macrophages (BMDM) that are recruited into the site. The primary tumor can secrete soluble factors and exosomes that reprogram resident macrophages in distant organs to become MAMs that are predominantly M2-activated, recruit BMDMs and help generate the pre-metastatic niche (73). Soluble factors (e.g., VEGF, TGFβ, TNFα, CXCL12) secreted from the primary tumor can activate lung cells to express S100A8/S100A9 and MMP-9 (74), and circulating CD11b+ myeloid cells are recruited to the lung in a VEGFR1-dependent manner (75). These MAMs and recruited BMDMs secrete VEGF and IL-1β, and help promote extravasation of DTCs at the secondary organ and their seeding through cross-talk involving CD44, integrins and endothelial cell adhesion molecules (13). Different techniques used to deplete macrophages help exemplify their important role during DTCs extravasation, survival, seeding and initial growth in the lung or bone (76, 77). However, the role that TAMs play during colonization of DTCs and in the process of escape from dormancy and metastatic outgrowth is still not fully understood.

Limited evidence suggests that the immune system, particularly MAMs, is important during initiation of the MET process and colonization, as well as in the regulation of dormancy and the metastatic outbreak. The metastatic outbreak is associated with inflammation, as patients with chronic inflammatory diseases (e.g., obesity, asthma, rheumatoid arthritis, psoriasis), increased serum levels of pro-inflammatory cytokines (e.g., IL-6) and presence of inflammatory infiltrate have an increased risk of metastasis (13). Similarly, surgical resection of the primary tumor, which is followed by a healing response, can enhance the escape from dormancy (13, 78). The presence of macrophages and the factors they secrete (e.g., CCL2, CCL5, IL-8) could also promote the escape from dormancy by activating the ERK signaling pathway (79, 80). In addition, interactions mediated through the binding of VCAM-1 to α4 integrins on DTCs induce Akt activation and promote DTCs survival (81). TNFα-induced expression of the IL-35 receptor IL12Rβ2 already on 4T1 mammary cancer cells in the primary tumor, rendered these cells more susceptible to the effects of IL-35 secreted from MAMs upon arrival at the lung metastatic site. This induced the JAK2/STAT6/GATA3 signaling, initiated their MET process and enhanced their colonization (82). In bone metastases, secreted pro-inflammatory cytokines including Parathyroid hormone-releasing peptide (PTHrP) stimulate osteoclasts to secrete RANK-L to promote bone degradation, and this releases dormant cells from the control of the bone microenvironment and promotes their proliferation and escape from dormancy (83). However, not all pro-inflammatory signals induce metastatic outgrowth. For example, secretion of IFNβ and IFNγ from TAMs and lymphocytes was shown to induce dormancy in DTCs by activating the IDO1/AhR/p27-dependent pathway (84, 85), and chemotherapy-induced secretion of IFNβ enhanced immune dormancy in an IRF7-dependent manner, drastically reducing the presence of MDSCs in the primary tumor and in the lungs of syngeneic mice (86). Interactions between the Growth Arrest Specific 6 (GAS6) factor produced by osteoblasts and the Axl tyrosine kinase receptor expressed by disseminating prostate cancer cells, mediated the TGFβ2 signaling pathway and promoted dormancy of the tumor cells in the bone marrow niche (87).

Different methods used to deplete macrophages demonstrated the importance of TAMs and MAMs in metastasis. In mouse models, removal of the spleen, which acts as a reservoir of myeloid cells, reduces metastasis (88). A recent study showed that TAMs are critical in a model of breast-cancer bone metastasis, as their ablation or inhibition resulted in reduced proliferation and metastasis (77). These TAMs originated mostly from monocyte-derived macrophages and not from bone resident macrophages, and their activation depended on their high IL-4R expression (77).

ECM composition and density could also play a role in determining dormancy or escape from dormancy, and TAMs and MAMS have been shown to take part in regulating this effect (50). For example, depletion of myeloid cells prevented M-MDSCs-induced deposition of Versican. Versican was shown to attenuate Smad2 and Snail levels, thus inhibiting EMT and promoting MET at the metastatic site (89). Collaboration between TAMs and cancer-associated fibroblasts (CAFs) that was initiated by systemic inflammatory signals, resulted in production of high levels of ECM proteins, including collagen, fibronectin and laminin, which increased ECM stiffness and generated a fibrotic MME (24). These changes protected the emerging DTCs from cytotoxic drugs and targeted therapies (24, 32). Neutrophils (and some macrophages) are usually associated with Neutrophil Extracellular Traps (NETs) that contain among other proteins the Neutrophil Elastase and MMP-9. These enzymes can degrade and remodel Laminin-rich ECM, and the cleavage products activate the α3β1 integrin pathway to promote proliferation and escape from dormancy (90).

Overall, the majority of evidence so far suggest that TAMs and MAMs promote MET and the escape from dormancy, but our mechanistic knowledge about the specific contribution of TAMs to the final step of the metastatic outgrowth is still very limited and merits more investigations.

## Targeting CD147 to Inhibit Metastasis

CD147 (also known as EMMPRIN or basigin) is a multifunctional transmembrane glycoprotein, with two extracellular Ig-like domains, that mediates interactions between tumor and stromal cells (91), and those interactions elevate the secretion of pro-angiogenic factors. CD147 is overexpressed in many types of tumors (91), and is elevated on metastatic cells (92–94) and in the serum of breast cancer patients with lymph node metastasis (95). CD147 is best known for its pro-angiogenic activity as an inducer of both VEGF and MMPs through homophilic interactions between tumor cells and stroma cells (mostly fibroblasts and macrophages). This homophilic interaction is localized to the N-terminus of the protein on the first of its two Ig-like domains (96, 97). Recently, we have identified a specific short epitope in the protein that is responsible for the induction of both VEGF and MMP-9 (98), and showed that CD147 is an important mediator of macrophage-tumor cell interactions, and its expression is increased when these cells are co-cultured *in vitro* (99). CD147 acts as a hub protein that binds to many protein partners and promotes the assembly of protein complexes (100), and therefore, is involved in mediating many functions (**Table 1**). For example, CD147 acts as a chaperone of the lactate transporters MCT-1 and MCT-4, to facilitate lactate efflux (126), which is critical in maintaining viability of tumor cells that rely mostly on glycolytic metabolism. CD147 also regulates Hyaluronan synthesis and can bind to the Hyaluronan receptor CD44, contributing to tumor cell invasiveness and chemoresistance (119, 135). Extracellular Cyclophilin, especially Cyclophilin A (CyPA) secreted by tumor cells, binds to its receptor CD147 to enhance tumor cell proliferation and survival and leukocyte chemotaxis and adhesion (105, 136). It was also suggested that by binding to CD147, CypA enhanced the localization of CD147 and CD44 complexes to lipid rafts and initiated a STAT3 signaling, thus activating Cyclin D1 and Survivin and promoting cell proliferation and survival (118). Additionally, CD147 has been linked to EMT, both as a mediator of TGFβ1 signaling leading to increased expression of the EMT transcription factors snail and slug (130, 131), and as a destabilizer of the interactions between E-cadherin and β-catenin (132). CD147 can also bind to the β1 integrins, especially α3β1 and α6β1 integrins, induce the FAK signaling pathway and promote invasion, MMPs secretion and metastasis (113, 137, 138). Invasiveness of malignant cells was associated with the increased expression of another binding partner - S100A9 (122, 139). Most importantly, CD147 activates the Wnt, the TGFβ1-Smad4, and/or the ERK1/2 pathways to enhance cell proliferation (101, 105, 140, 141), potentially implicating CD147 in the regulation of metastasis and escape from dormancy. Collectively, these studies suggest that CD147 acts as a scaffold protein that promotes the assembly of many proteins into one or more signaling complexes that enhances cell proliferation, metabolism, angiogenesis, invasiveness, EMT and survival. All these processes are necessary for tumor metastasis, and the increased proliferation and angiogenic switch promote the escape from cellular and angiogenic dormancy. These data place CD147 as an attractive candidate for targeting.

**Table 1:** The multifunctional CD147 protein acts as a hub protein that binds many protein partners, thus promoting processes that could lead to escape from dormancy.

Process required for metastasis/dormancy	CD147 Binding Protein Partner	Signaling pathways activated or implicated, and resulting products	References
Proliferation	Mechanism unclear. Candidate partners: Smad4, gasdermin D	Wnt/β-catenin, Notch1 and PI3K/Akt pathways, regulating cell cycle proteins	(101–104)
Proliferation, cytokine secretion, adhesion	Extracellular CyP A/B	ERK, NF-κB, PI3K	(105–108)
Angiogenic switch	CD147 (homophilic interaction)	ERK, PI3K signaling	(96, 97)
		VEGF, MMP-9	(109–112)
Adhesion to ECM proteins	integrins (α3β1, α6β1)	PI3K, FAK/paxillin, Ca^+2^ signaling, cytoskeletal reorganization	(113–117)
Invasiveness	Hyaluronan, CD44, CypA	ERK, PI3K, STAT3 signaling	(118–121)
Migration, pro-inflammatory cytokines	Calprotectin (S100A8/S100A9)	NF-κB	(122, 123)
Drug resistance	P-gp/ABCB1, CD44, ABCG2	ERK	(120, 124, 125)
Metabolism, lactate efflux	MCT-1/4, facilitates lactate efflux	Not yet known	(126–129)
EMT	CD147 overexpression, affected by TGFβ-mediated signaling	TGFβ, Wnt/β-catein pathway, Snail, Slug	(130–134)

Targeting CD147 by siRNA revealed much about the functions of the protein. Reduced CD147 expression inhibited the expression of MMP-9 and MMP-2 and markedly reduced the invasion of PC-3 prostate cancer cell line (142) and in the HCC cell line FHCC-98 (143). In the breast cancer MCF7 cell line it reduced both MMP-9 and VEGF expression, as well as cell proliferation, migration and invasion (93). Silencing CD147 in the leukemic U937 cell line inhibited cell viability and proliferation, and was associated with downregulation of Cyclin D1 and Cyclin E, and with increased sensitivity to the doxorubicin/Adriamycin (102). Silencing CD147 expression in the human malignant melanoma cell line A375 resulted in morphological changes, where adhesion to the collagen-coated plates, but not to fibronectin-, fibrinogen- or laminin-coated plates, was decreased, and integrin β1 expression was re-localized from the plasma membrane to the cytoplasm (114). *In vivo*, tumors generated with the HCC Hepa1-6 cell line that was silenced for CD147 expression, exhibited reduced tumor size and increased T cell infiltration and were sensitized to their cytotoxicity (144), suggesting that CD147 mediated escape from immune surveillance. Xenografts of the human HNSCC cell line FaDu cells demonstrated decreased tumor growth, reduced proliferation and reduced levels of MMP-9 and VEGF after CD147 was knocked down (145), and tumors generated with the mouse CD147-KD P388D1 cells resulted in smaller tumors, inhibited MMP-11 levels, and reduced ability to migrate to lymph nodes (146). These results implicate CD147 in the induction of MMPs, invasion, adhesion and proliferation, which are all critical during different stages of metastasis.

Most antibodies that were developed against CD147 remained in the pre-clinical stages. For example, a chimeric anti-CD147 antibody (CNTO3899) directed against the extracellular domains of CD147 inhibited tumor growth in a SCC-1 xenograft model of HNSCC, and even more so when combined with radiotherapy (147). Another murine monoclonal antibody (clone 1A6) that targeted human CD147 inhibited tumor growth in orthotopic model of human pancreatic cancer (148) and cutaneous squamous cell carcinoma (SCC) (149), and demonstrated reduced proliferation and migration of the tumor cell lines. Our group developed an antibody against the above-mentioned epitope in CD147, and demonstrated reduced tumor growth in two subcutaneous models of colon and renal cell carcinomas, and reduced metastasis in an orthotopic model of 4T1 mammary gland cancer (98). We also showed that the antibody reduced metastasis in a MDA-MB-231 orthotopic model (unpublished data). Moreover, active vaccination with a modified peptide against the same epitope resulted in inhibition of tumor growth and lung metastases, and prevented recurrence when challenged with repeated injections of the tumor cells (150). We showed that targeting CD147 with the antibody changed the TME by inducing a necroptotic death, which released dsRNA to the TME and shifted macrophage activation to promote anti-cancer immunity (151).

CD147 is highly expressed in hepatocellular carcinoma (HCC), which is a malignancy characterized by rapid progression, poor prognosis, and frequent tumor recurrence despite surgical treatment modalities (152, 153). In order to target CD147 in HCC, the anti-CD147 monoclonal antibody HAb18 was generated in mice immunized with hepatocellular carcinoma cells and cleaved with pepsin to remove the Fc fragment. The resulting F(ab’)_2_ fragment that targets the EC-1 extracellular domain of CD147 was conjugated to iodine (^131^I) and named Metuximab/Licartin (154, 155). In phase I/IIc clinical trials, radioimmunotherapy with ^131^I-metuximab was directed into the tumor and found to be safe and effective (154, 155), and subsequently was approved for clinical therapy of primary HCC by China State Food and Drug Administration. Overall, meta-analysis demonstrated that ^131^I-metuximab in combination with transcatheter arterial chemoembolization (TACE) or with radiofrequency ablation (RFA) exhibited high specificity and efficiency in killing tumor cells and extending survival (156–158). The use of Metuximab showed a decrease in recurrence and increased survival rate of treated patients after resection followed by orthotopic liver transplantation or in combination with radiofrequency ablation (152, 157), suggesting that the antibody can target residual metastatic cells. Mechanistically, it was suggested that the two components of ^131^I-metuximab - the radioactive moiety and the antibody fragment - synergize to kill HCC cells, inhibit their proliferation and reverse the EMT process (159). The same Chinese group later developed another human-mouse chimeric antibody that recognized the EC-2 domain of CD147 named metuzumab (160), and showed that it was efficient in inhibiting tumor growth in xenograft models of lung cancer human in SCID mice, and could sensitize tumor cells to chemotherapy (161). However, metuzumab has not yet reached clinical trials.

## Conclusion

Cancer recurrence remains a major problem in cancer treatment, and is attributed to the awakening of dormant DTCs in local or remote organs. The development of metastasis is a complex, multi-step process whose molecular mechanisms are not yet fully elucidated, which is intimately associated with macrophages and other immune cells. Therefore, finding proteins that are closely implicated in the decision of DTCs to escape dormancy might provide new therapeutic targets that will prevent tumor recurrence, and it is reasonable to consider proteins that are involved in these immune interactions.

CD147 had been implicated in driving the EMT process, and has been shown to take part in the regulation of angiogenesis, proliferation, migration, invasion, and chemoresistance, all of which are processes critically required by the DTCs that escapes dormancy. Evidence that directly place CD147 as a participant in the MET process are not yet available, but its involvement in proliferation certainly suggests that this is the case. CD147 is also a mediator of tumor cell-macrophage interactions, so targeting it might disrupt those interactions and delay or prevent the escape from dormancy. Thus, targeting CD147, alone as a monotherapy or better yet in combination with radiotherapy, chemotherapy, or other immunotherapies, could potentially inhibit or prevent all processes that are essential for the escape from dormancy simultaneously.

## Author Contributions

The author confirms being the sole contributor of this work and has approved it for publication.

## Funding

MR is supported by the Israel Science Foundation (ISF) grant number 2607/20 and the Israel Cancer Association (ICA) grant number (20210046).

## Conflict of Interest

MR is an inventor of a patent (US Grant US9688732B2, EP application EP2833900A4) related to peptide vaccination approach against CD147 mentioned in the manuscript.

## Publisher’s Note

All claims expressed in this article are solely those of the authors and do not necessarily represent those of their affiliated organizations, or those of the publisher, the editors and the reviewers. Any product that may be evaluated in this article, or claim that may be made by its manufacturer, is not guaranteed or endorsed by the publisher.
